# How mammalian piRNAs instruct de novo DNA methylation of transposons

**DOI:** 10.1038/s41392-020-00294-5

**Published:** 2020-09-07

**Authors:** Zhiqing Li, Xiaoyin Tang, En-Zhi Shen

**Affiliations:** 1 Key Laboratory of Growth Control and Translational Research of Zhejiang Province, School of Life Sciences, Westlake University, Hangzhou, Zhejiang China; 2 Institute of Biology, Westlake Institute for Advanced Study, Hangzhou, Zhejiang China; 3 Fudan University, Shanghai, China; 4 College of Life Sciences, Zhejiang University, Hangzhou, Zhejiang China

**Keywords:** Epigenetics, Epigenetic memory

A recent study published in *Nature* by Zoch et al. identifies a novel MIWI2-associated protein SPOCD1 and shows its requirement for piRNA-guided young transposable elements (TEs) methylation and silencing.^1^ This research provided the first mechanistic insights into how piRNA directs de novo DNA methylation in male mammals (Fig. 1).

The germline plays a crucial role in the continuation of sexually reproducing species via producing gametes.^2^ TEs are mobile genetic elements, which greatly threaten the development and maintenance of a functional germline and therefore must be restrained. Fortunately, germ cells employ a specialized small RNA silencing pathway—PIWI-interacting small RNAs (piRNAs) and their PIWI protein partners to eliminate this threat through epigenetic silencing mechanisms.^3^ The nuclear PIWI protein MIWI2, together with associated piRNAs has been proposed to active TE loci by base pairing to nascent transcripts and execute DNA methylation,^4^ but the mechanism remains largely unknown.

**Fig. 1:**
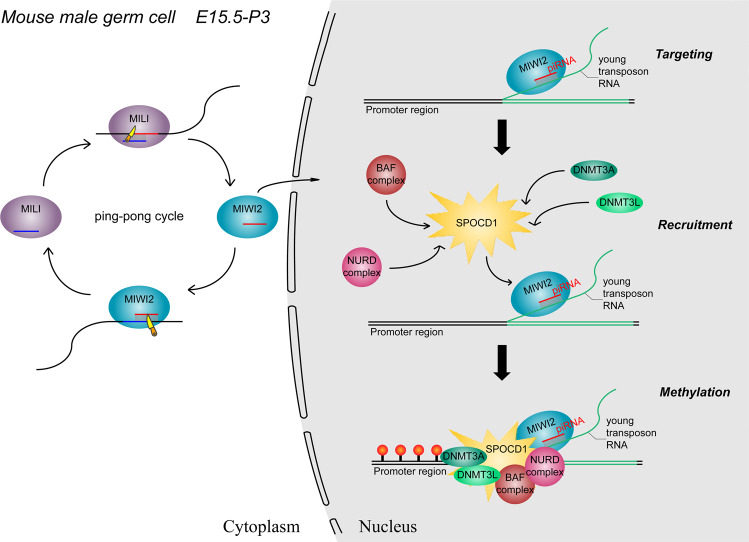
Schematic model of piRNA-guided de novo DNA methylation of transposons in mouse male germline in a time window from embryonic day 15.5 (E15.5) to postnatal day 3 (P3). MIWI2 engages in the ping–pong cycle with MILI, leading to the initiation of effector piRNA production. Loading of MIWI2 with resulting effector piRNAs induces its translocation into the nucleus. The MIWI2/piRNA complex then targets young transposon RNA through high complementarity of base-pairing, which subsequently licences the recruitment of SPOCD1 and the associated DNA methylation and chromatin remodeling machinery, resulting in targeted DNA methylation of promotor elements upstream of transposon loci

To explore the downstream mechanisms of how MIWI2/piRNA instructs de novo TE methylation, Zoch et al. first defined the interactome of MIWI2 in foetal gonocytes. They generated a *Miwi2*^*HA*^ allele by fusing the HA epitope tag to the N-terminus of endogenous fully functional MIWI2 and then used anti-HA immunoprecipitation coupled with quantitative mass spectrometry (IP–MS) to do proteomics analysis. This approach identified 28 MIWI2-associated proteins. To determine the nuclear proteins in directing de novo TE methylation, authors established two criteria. First, expression pattern of the gene should be restricted to the period of de novo methylation. Second, the protein encoded by this gene should be located in the nucleus. Only a single gene *Spocd1* meets these two criteria. These data indicated that a novel MIWI2 interactor SPOCD1 may be a promising candidate for the execution of nuclear MIWI2 function.

In order to decipher the potential function of SPOCD1 in the piRNA pathway, authors engineered Spocd1 mutation (*Spocd1*^−^) in mice. They observed that *Spocd1*^−*/*−^ male were infertile and lacked spermatozoa in the epididymis. The *Spocd1*^−*/−*^ testes were substantially smaller than their wild-type counterparts. In addition, the expression of long interspersed nuclear element-1 (LINE1) and intracisternal A-particle (IAP), which are normally silenced in adult wild-type testes, can be detected in the testes of adult *Spocd1*^*−/*−^ mutant. Staining of γ-H2AX, a marker of the persistence and strength of the DNA damage response, revealed the extensive unrepaired double-stranded breaks in *Spocd1*^*−/*−^ meiocytes. Taken together, these phenotypes implied that SPOCD1 is required for spermatogenesis and transposon repression, and are consistent with the ones caused by the loss of piRNA pathway activity.

piRNA pathway contributed to establish genomic methylation patterns on IAP and LINE1 elements.^5^ Zoch et al. next set out to determine whether SPOCD1 is essential for piRNA-directed de novo DNA methylation. Whole genome methylation sequencing (Methyl-seq) were performed using genomic DNA from wildtype, *Spocd1*^−*/−*^ and *Miwi2*^−*/*−^ P14 spermatogonia. Demethylation was detected specifically in IAPEy and MMERVK10C as well as the young LINE1 families but not collective transposons in *Spocd1*^*−/*−^ spermatogonia, which was consistent with the demethylation pattern of Miwi2-deficiency. Importantly, Metaplots further indicated that loss of methylation occurs specifically at TE promoter elements in *Spocd1*^*−/−*^ spermatogonia, which corresponds to characteristics of piRNA-directed methylation. Collectively, SPOCD1 plays a role in piRNA-guided DNA methylation.

To further explore the exact role of SPOCD1 in piRNA pathway, either in piRNA biogenesis or alternatively in downstream of MIWI2 function, Zoch et al. first analysed expression of small RNAs in *Spocd1*^+/−^ and *Spocd1*^−*/−*^ E16.5 foetal testes. No major changes were detected in piRNA length distribution, annotation of mapped piRNAs, relative piRNA counts, piRNA amplification, or their mapping to TEs. Supportively, MIWI2 exhibited the normal localization in the nucleus in the absence of *Spocd1*, confirming that SPOCD1 has no effect on the piRNA processing and implying its role in the downstream of nuclear MIWI2 function. An alternative nuclear function could be acting as a transcription factor essential for either transposon or gene expression. However, RNA-seq data from E16.5 foetal gonocytes ruled out this possibility. To further investigate how SPOCD1 contributes to methylation of TEs, authors then performed anti-HA IP–MS from *Spocd1*^*HA/+*^ E16.5 foetal testes to dig out interactome of SPOCD1-HA. Components of the de novo methylation machinery such as DNMT3L and DNMT3A, as well as several components of the repressive chromatin remodeling NURD and BAF complexes were detected. Importantly, using the same stringent association criteria, they found that DNMT3L, NURD and BAF components can also be detected in the MIWI2 IP.

In summary, these studies together show that, probably by recruiting DNA methyltransferases (DNMT3L and DNMT3A) and chromatin remodeling complexes (NURD and BAF), a novel nuclear protein SPOCD1 serves as an executor for MIWI2/piRNA-mediated de novo DNA methylation of mammalian young transposons. This work will advance our basic understanding of piRNA function in fertility and epigenetic inheritance, and may guide our understanding of how piRNA pathway directs DNA methylation of TEs in human beings.

## References

[1] Zoch A (2020). SPOCD1 is an essential executor of piRNA-directed de novo DNA methylation. Nature.

[2] Tang WW, Kobayashi T, Irie N, Dietmann S, Surani MA (2016). Specification and epigenetic programming of the human germ line. Nat. Rev. Genet..

[3] Ozata DM, Gainetdinov I, Zoch A, O’Carroll D, Zamore PD (2019). PIWI-interacting RNAs: small RNAs with big functions. Nat. Rev. Genet..

[4] Carmell MA (2007). MIWI2 is essential for spermatogenesis and repression of transposons in the mouse male germline. Dev. Cell.

[5] Aravin AA (2008). A piRNA pathway primed by individual transposons is linked to de novo DNA methylation in mice. Mol. Cell.

